# Prisoner's Dilemma in Cancer Metabolism

**DOI:** 10.1371/journal.pone.0028576

**Published:** 2011-12-14

**Authors:** Irina Kareva

**Affiliations:** 1 Mathematical, Computational Modeling Sciences Center, Arizona State University, Tempe, Arizona, United States of America; 2 School of Human Evolution and Social Change, Arizona State University, Tempe, Arizona, United States of America; Arizona State University, United States of America

## Abstract

As tumors outgrow their blood supply and become oxygen deprived, they switch to less energetically efficient but oxygen-independent anaerobic glucose metabolism. However, cancer cells maintain glycolytic phenotype even in the areas of ample oxygen supply (Warburg effect). It has been hypothesized that the competitive advantage that glycolytic cells get over aerobic cells is achieved through secretion of lactic acid, which is a by-product of glycolysis. It creates acidic microenvironment around the tumor that can be toxic to normal somatic cells. This interaction can be seen as a prisoner's dilemma: from the point of view of metabolic payoffs, it is better for cells to cooperate and become better competitors but neither cell has an incentive to unilaterally change its metabolic strategy. In this paper a novel mathematical technique, which allows reducing an otherwise infinitely dimensional system to low dimensionality, is used to demonstrate that changing the environment can take the cells out of this equilibrium and that it is cooperation that can in fact lead to the cell population committing evolutionary suicide.

## Introduction

Cancer can be viewed as a long evolutionary process within one person. Even in the cases of most severe DNA damage, such as was experienced by the survivors of atomic bombing in Hiroshima and Nagasaki, it is not until the 50 s that one could observe dramatically increased cancer incidence [1]. Damaged cells, whatever properties they may have acquired, need to survive and proliferate in the tissue, competing with somatic cells for space and nutrients.

As the primary tumor increases in size, the cells outgrow their blood supply, thus also losing access to oxygen. This leads to cells in hypoxic areas switching from aerobic metabolism to glycolysis, a mode of glucose metabolism that is less energetically efficient, yielding 2 ATPs instead of approximately 30, but that is faster and, most importantly, unrestricted by oxygen. However, tumor cells often continue metabolizing carbon glycolytically even in the areas of ample oxygen supply [2]–[4]. This has become known as Warburg effect, named after a German biochemist Otto Warburg, who was the first to observe it over fifty years ago [5]. This choice of metabolic strategy does not come from loss of function of mitochondria – it has been verified that a vast majority of tumor cells have completely functional mitochondria [6], and the damage that might be occurring is reversible [7].

From the point of view of natural selection, it has been hypothesized that, although glycolysis is energetically inefficient, lactic acid that is secreted as its by-product becomes toxic to healthy tissues, thus making glycolytic cells better competitors at a cost of being efficient consumers [8], [9]. However, a single cell is not likely to secrete enough lactic acid to cause significant changes in its microenvironment, i.e., it cannot provide enough “public goods” to benefit everyone [10]. The core population of glycolytic cells needs to be large enough to gain this competitive advantage. Proposed here is a game-theoretical approach for addressing the question of how such a population could arise.

### Game theory in cell metabolism

As advantageous as glycolysis may be to cancer cells as a group, one glycolytic cell is not enough to generate enough lactic acid to become a successful competitor. Enough cells need to choose this metabolic strategy in order for the group as a whole to receive the competitive advantage. However, it is not in the interest of each individual cell to metabolize carbon glycolytically if all other cells metabolize it aerobically. It would not secrete enough lactic acid to successfully compete with them and at the same time, it would get nearly 15 times less energy.

In this framework, the problem becomes a version of multi-player prisoner's dilemma. There are two metabolic strategies: aerobic, which yields 30 ATPs per glucose and no lactic acid, and glycolytic, which yields 2 ATPs per glucose but yields some lactic acid. The amount of lactic acid generated by just one glycolytic cell is insignificant to cause any damage to somatic cells. Lactic acid secreted by several cells is enough to shift energetic payoffs, which could in part be due to not only decrease in competition but also to the fact that intracellular stores of nutrients of the cells can be recycled and thus used up by neighboring cells [11], [12]. For illustration we currently assume 2 players but in fact many more would need to cooperate to get this “public goods” effect [10]. This becomes a game of prisoner's dilemma if the payoff for both cells is greater when they both choose the glycolytic strategy, i.e., if [30 ATP<2+toxicity+reduced competition]. In this case, the aerobic-aerobic equilibrium is in fact a stable equilibrium of this game, i.e., no cell has an incentive to unilaterally change its metabolic strategy [13], [14]. So, from the point of view of metabolic activity, one can argue that aerobic cells are in fact at an evolutionarily steady state [15], and so the tissue cannot be “invaded” by glycolytic clones.

Nevertheless, “glycolytic invasions” do happen as the Warburg cells migrate out of the primary tumor into the new environment composed primarily of aerobic cells, where they theoretically should have no advantage in persisting to metabolize glucose glycolytically. One explanation for this effect could be that they in fact migrate out in groups large enough to generate enough lactic acid for everyone to receive sufficient “public goods” benefit.

Another (perhaps complementary) explanation comes from invasion ecology, and particularly from the work of David Tilman, who argued that invasions of exotic species are largely facilitated when there are excess resources available in the target habitat for the invaders to utilize [16], [17]. In the case of aerobic and glycolytic cells, if there are enough resources in the environment into which the cell migrates out to, then a glycolytic cell will no longer have to care about its metabolic inefficiency. That is, from the point of view of payoffs of each metabolic strategy, if the environment, in which the players interact, changes sufficiently, glycolytic invasion becomes possible.

To test this hypothesis, a mathematical model is proposed. The change in the composition of the population of cells that differ by their choice of metabolic strategy (glycolysis vs oxidative phosphorylation) in response to increased carbon inflow is tracked using a system of ordinary differential equations. In the model, the growth of aerobic cells is restricted both by carbon and oxygen, while glycolytic cells are restrained only by carbon. The effects of changes in oxygen availability, glucose uptake rates, natural cell death rates, cell growth rates, as well as the initial composition of the cell population are evaluated.

## Materials and Methods

### Model Description

Suppose that each cell is characterized by a value of parameter 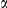, which represents the proportion of total carbon that is used aerobically, thus effectively leaving 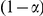 proportion of total carbon for consumption through glycolysis; 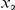 then denotes a set of all cells that are characterized by a fixed heritable value of parameter 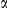. The total population size is then taken to be 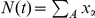 if the number of possible values of 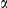 is finite and 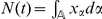 if it is infinite.

Glycolysis is less metabolically efficient and is limited only by glucose supply, denoted by 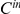; aerobic metabolism is more efficient but is limited both by carbon availability 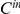 and by oxygen supply, which is accounted for with parameter 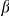. Each cell 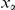 is thus characterized by its own intrinsic value of 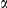, allowing to model population heterogeneity with respect to metabolic strategy.

There are two types of carbon that are taken into account in the model: extracellular carbon and intracellular carbon. Extracellular carbon 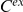 is replenished in the tissue microenvironment through blood inflow and also is recycled from intracellular stores of cells that have died [11], [12]. It is consumed by the cells, becoming intracellular carbon, based on differences in concentration between 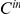 and 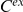. Different cells can consume carbon at different rates: glycolytic cells get less energy per one molecule of glucose, but their rate of carbon uptake is much greater due to upregulation of glucose transporters in the cell membrane [18]. This is accounted for by the parameter 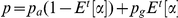. The consumed extracellular carbon is then metabolized by the cells; the higher efficiency of metabolism by aerobic cells is accounted for by the parameter 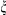.

Taking into account all of these assumptions, the model becomes System
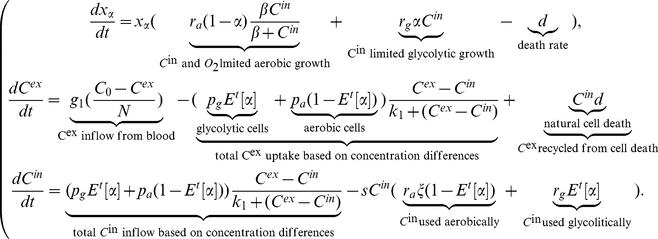
(1)


**Figure 1:**
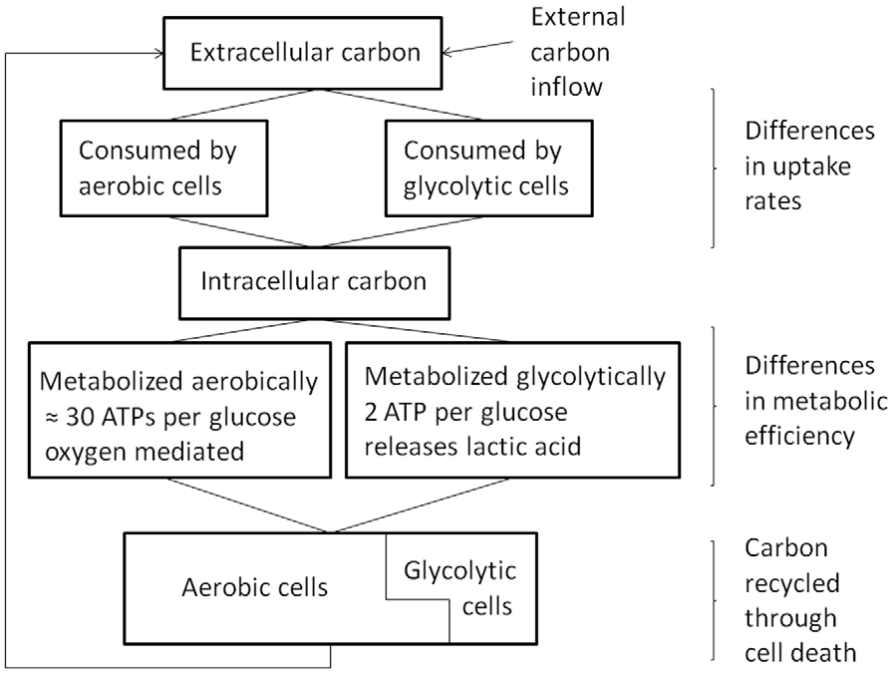
Schematic diagram of the process described in System (1).

A detailed model derivation is given in Appendix S1. The summary and description of all parameters is given in Table 1, and the general overview of the proposed mechanism is given in Figure 1.

**Table 1:** Summary of variables and parameters used throughout the paper.

Variable/Parameter	Meaning	Range
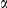	proportion of glycolytic cells	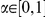
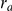	growth rate of aerobic cells	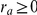
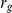	growth rate of glycolytic cells	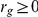
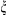	scaling constant to account for higher efficiency of aerobic metabolism (2 ATP vs 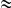 30 ATP)	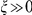
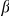	oxygen availablity (normal blood oxygen is 20%, hypoxia occurs around 2–5%)	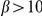
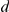	natural cell death rate	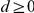
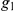	rate of resource consumption	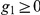
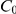	rate of external carbon inflow (normal carbon concentration in the blood is 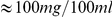 )	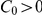
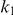	saturation constant for carbonuptake by the cells	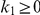
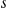	scaling constant for how muchcarbon is metabolized by cells	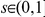
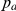	rate of carbon uptake by aerobic cells	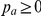
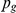	rate of carbon uptake by glycolytic cells	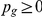
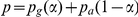	lower boundary value of parameter 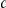	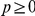
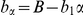	toxicity induced cell mortality	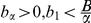

**Figure 2:**
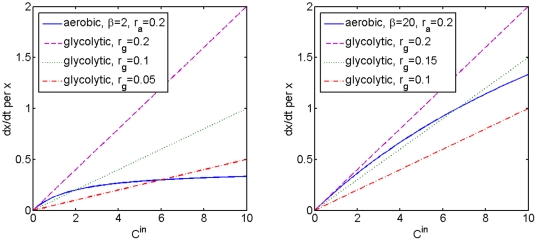
Relative positions of growth rates for aerobic and glycolytic cell clones. Growth rates for aerobic (
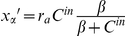
, solid blue line) and glycolytic (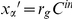, dashed lines) cell clones are compared for different initial states of the microenvironment (amount of resource 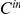 and amount of oxygen 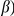 and different relative intrinsic growth rates 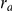
and 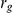. One can see that different clone types have higher fitness relative to each other depending on carbon (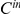) and oxygen (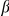) availability and the values of intrinsic parameters 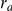 and 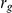.

Each cell clone 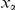 tries to maximize its fitness by metabolizing glucose either aerobically or glycolytically. Depending on initial population composition, on intrinsic growth and death rates, and the amount of carbon available, the clones are selected depending on which metabolic strategy maximizes their overall growth rate per cell, reflected through the value of 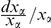. Relative positions of the two growth curves with respect to resource availability are shown in Figure 2.

### Modeling population heterogeneity

In a heterogeneous population, where each cell is characterized by its own value of parameter 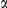, the mean number of glycolytic clones 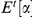 is a dynamic variable that can change over time. Therefore, the composition of a heterogeneous population of cells will also change as a result of the dynamics of other variables and will be different depending on initial conditions, parameter values, as well as the initial distribution of the clones within the population. (Note: in the current formulation, System (1) is an infinitely-dimensional system of ODEs. However, it can be reduced to a finitely-dimensional system of equations through addition of two keystone equations. The details of the transformation are described in Appendix S2; further references on the method can be found in [19])

System (1) was solved numerically using Matlab R2010a in such a way as to evaluate, how the composition of the population, tracked through 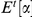, changes over time in response to increasing inflow of extracellular carbon, achieved through systematic increase of parameter 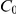 (external carbon inflow). The changes in 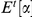 in carbon-rich environments were also evaluated with respect to changes in oxygen levels (parameter 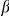), glucose uptake rates (changing relationship between parameters 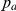 and 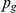), growth rates (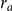 and 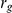) and natural death rates (parameter 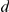).

## Results

The initial distribution of clones within the population was taken to be truncated exponential with parameter 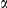 restricted to the interval 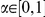, and skewed towards 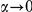, i.e., such that the vast majority of cells in the initial population are aerobic. This is to account for the differences in access to the oxygen and nutrients as a result of slight variations in distance from the blood vessels. Initial conditions and parameter values used for calculating numerical solutions are summarized in Table 2, unless indicated otherwise.

**Table 2:** Sample parameter values.

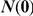	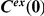	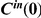	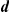	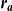	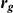	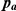	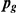	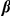	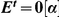	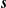	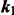	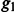
10.0	10.6	1.3	0.03	0.2	0.22	0.1	0.2	15	0.056	0.2	1.1	1.0

The results of all the calculations are presented using four types of graphs. The first type of graph depicts the changes in the proportion of glycolytic cells in the population 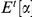 over time under variation of parameters that represent intrinsic properties of cells (proliferation, death, resource uptake rates, etc). On the second type of graph, external carbon inflow 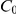 is varied and the value of 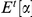 is recorded at 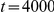 as the values of intrinsic parameters are varied. This is done to uniformly measure the effects of changes in external factors (nutrient availability) on glycolytic expansion; time point 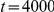 is chosen arbitrarily. The third type of graph is a 3-dimensional representation of how 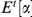 changes over time under different values of 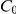. Finally, the fourth type of graph depicts the change in the distribution of clones with respect to strategy choice, over time.

**Figure 3:**
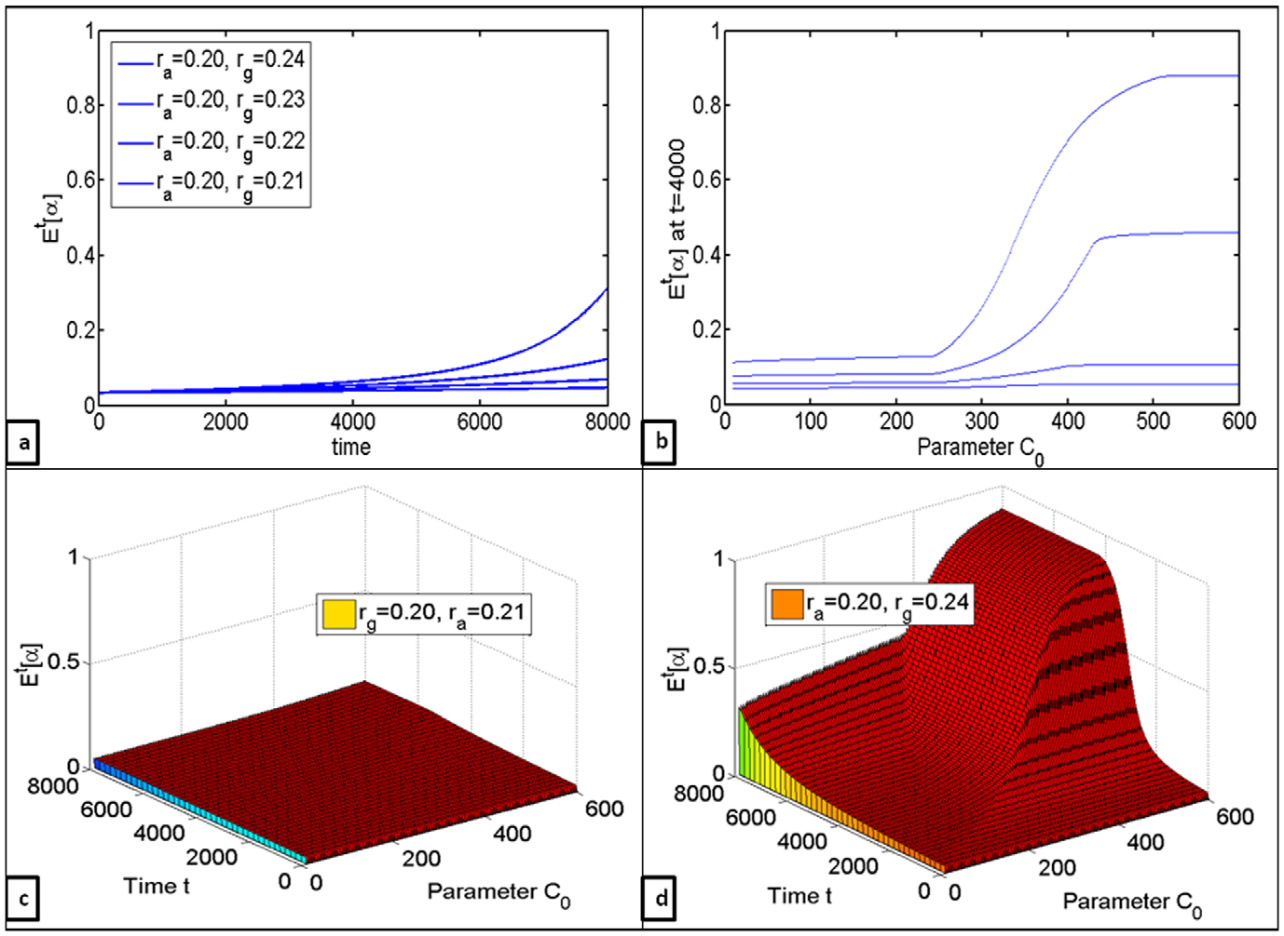
Quantifying the effects of differences in growth rates of aerobic and glycolytic cell clones. (a) Changes in the mean number of glycolytic cells 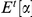 over time for 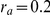, 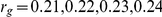 (b) 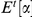 at 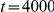 for 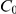 varied from 5 to 600, evaluated for 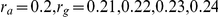 (c) Changes in 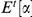 over time with respect to differences in 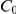 for 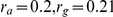 (d) Changes in 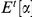 over time with respect to differences in 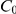 for 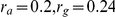.

At first the effects of changes in intrinsic growth rates were evaluated (see Figure 3). It can be observed that while, naturally, higher growth rates of anaerobic cells will always lead to increased proportion of glycolytic cells in the population (Figure 3a), increases in the rates of external carbon inflow 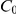 accelerate this process dramatically (Figure 3b–d).

**Figure 4:**
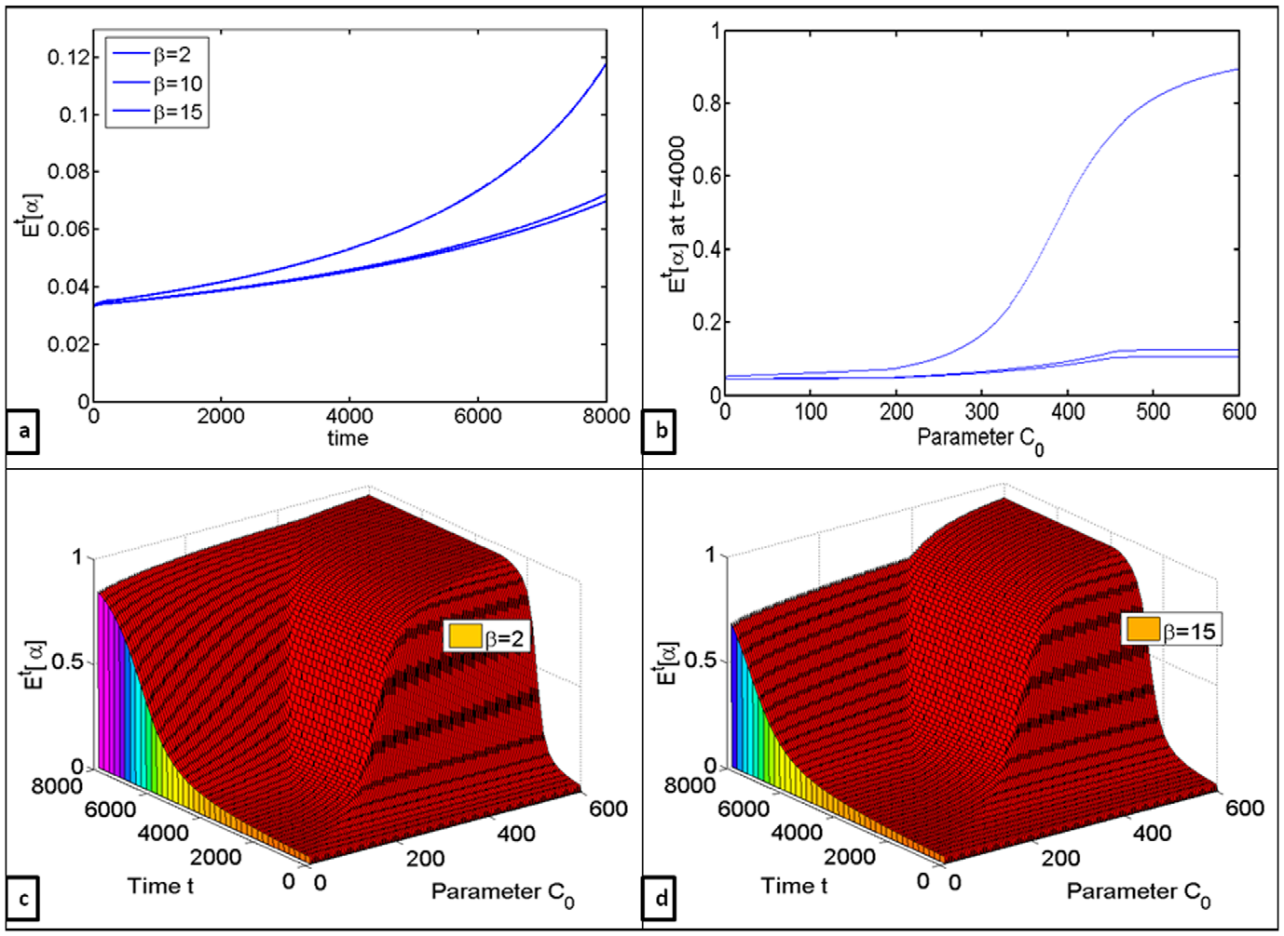
Quantifying the effects of oxygen availability on the growth of aerobic and glycolytic cell clones. (a) Changes in the mean number of glycolytic cells 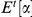 over time for 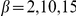 (b) 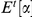 at 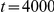 for 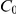 varied from 5 to 600, evaluated for 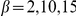 (c) Changes in 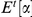 over time with respect to differences in 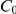 for 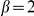 (d) Changes in 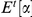 over time with respect to differences in 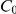 for 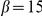.

The effects of oxygen availability, accounted for with parameter 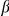, were evaluated in Figure 4, and in particular, the question of whether oxygen deprivation will have more or less effect on glycolytic expansion than increased carbon inflow. As anticipated, lower 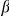 resulted in faster growth of glycolytic cells (Figure 4a). However, increases in carbon inflow resulted in nearly as much of glycolytic expansion as was caused by oxygen deprivation (Figure 4b–d), which suggests that under nutritionally favorable conditions benefits of glycolysis do indeed outweigh its drawbacks.

**Figure 5:**
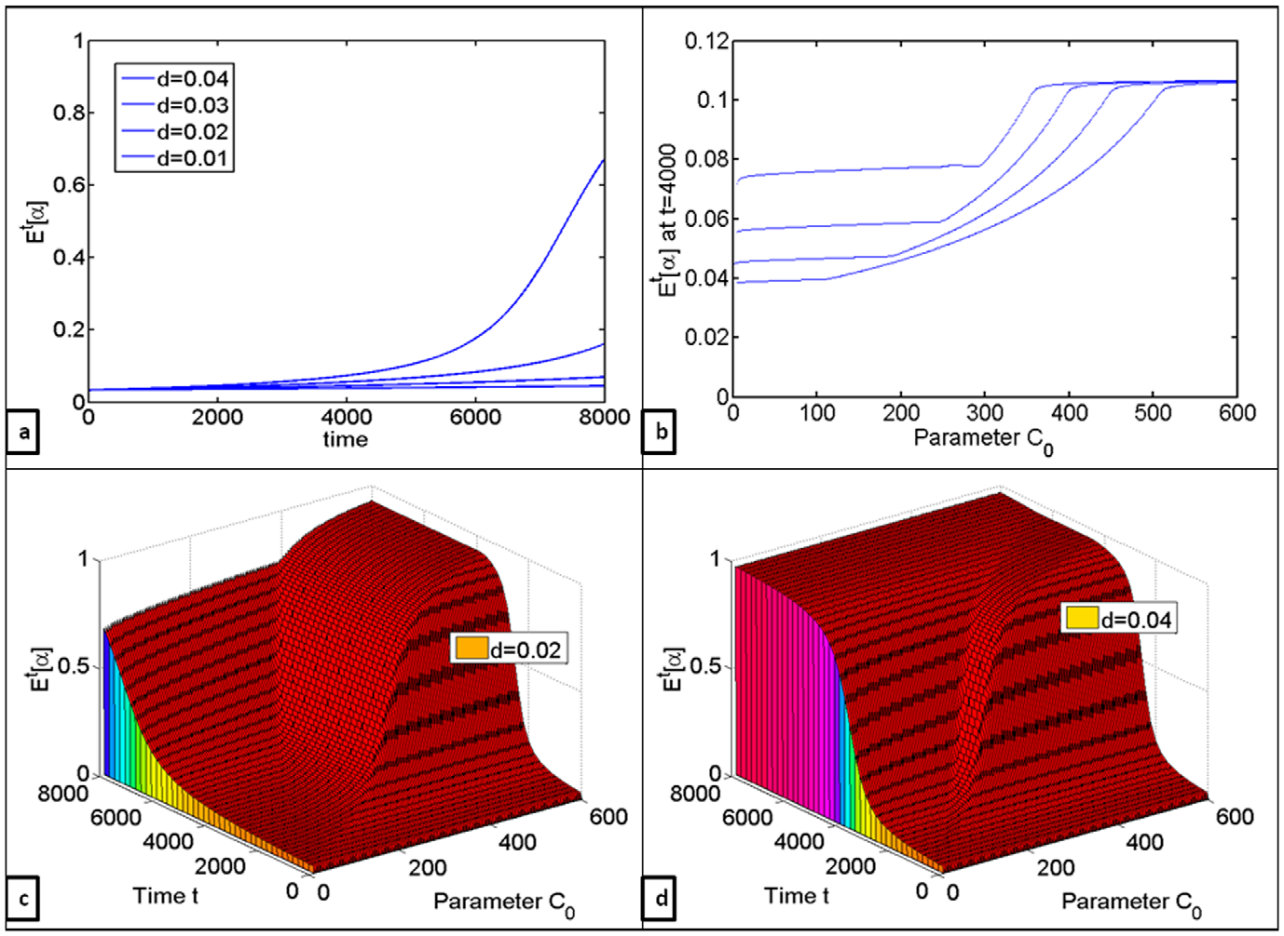
Quantifying the effects of natural death rates on the changes in proportion of glycolytic cell clones in the population. (a) Changes in the mean number of glycolytic cells 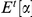 over time for (b) 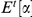 at 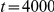 for 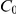 varied from 5 to 600, evaluated for 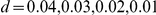 (c) Changes in 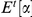 over time with respect to differences in 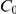 for 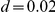 (d) Changes in 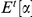 over time with respect to differences in 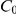 for 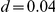.

Next, the effects in changes of natural cell death rates were evaluated. Interestingly, decreasing the value of parameter 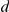 actually slowed down glycolytic expansion (Figure 5). That is, lower death rates are in fact less advantageous for glycolytic cells at this stage of tumor development. This effect could be due to the fact that higher cell death rates imply higher cell turnover within the population, thus actually speeding up the selective process. Lower death rates on the contrary cause a delay in the progression of the evolutionary process.

**Figure 6:**
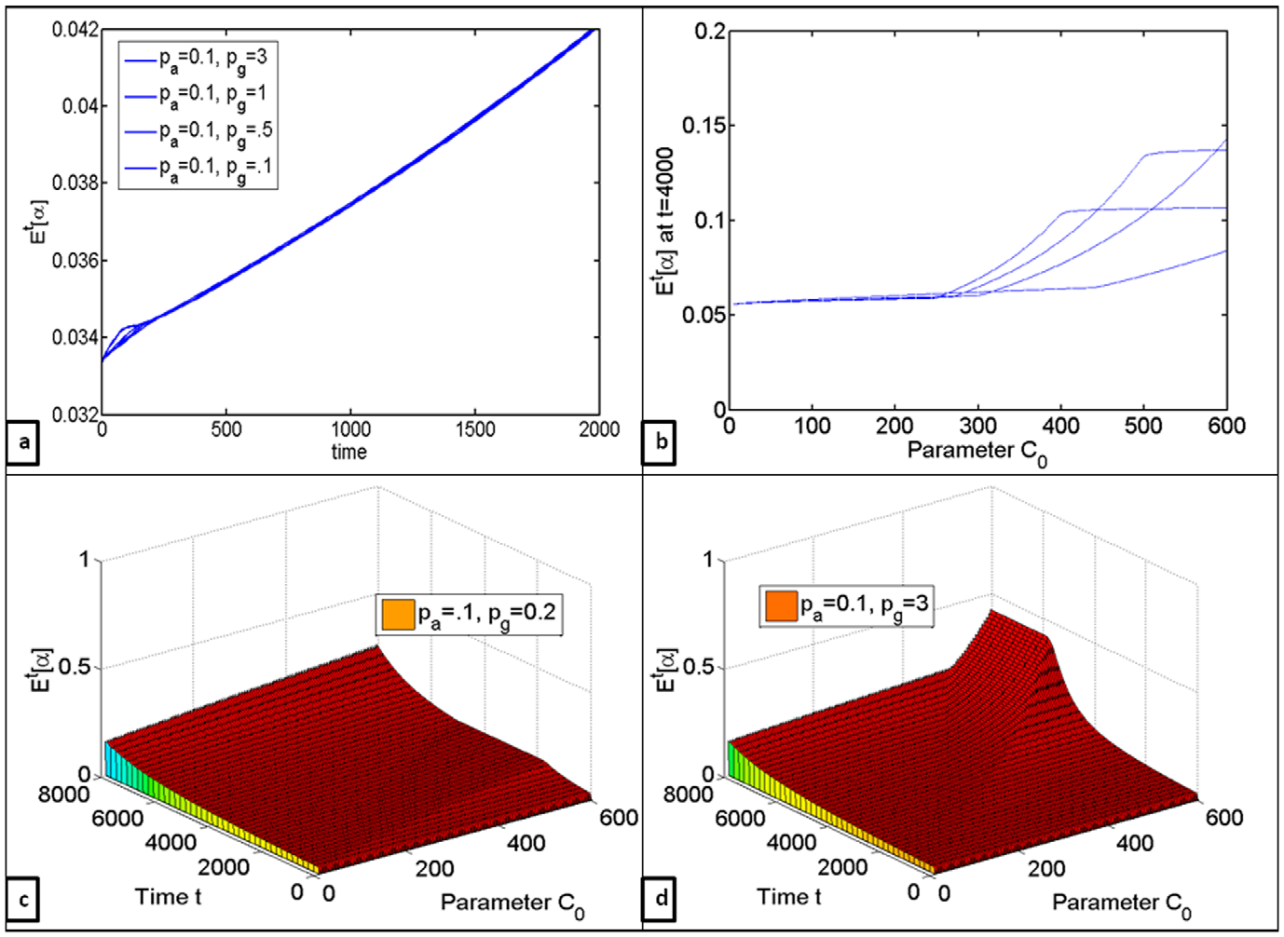
Quantifying the effects of differences in resource uptake rates on the changes in proportion of glycolytic cell clones in the population. (a) Changes in the mean number of glycolytic cells 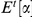 over time for 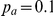, 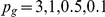 (note the scale on y-axis) (b) 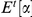 at 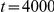 for 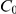 varied from 5 to 600, evaluated for 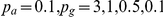
(note the scale on y-axis) (c) Changes in 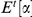 over time with respect to differences in 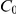 for 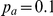, 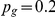 (d) Changes in 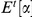 over time with respect to differences in 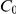 for 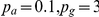.

The effects of differences in nutrient uptake rates were evaluated, since cancer cells have been observed to consume extracellular carbon much quicker than aerobic cells, with uptake rates between the two types differing as much as 10–20 times [18]. The question here was whether upregulation of glucose transporters would be enough to give cancer cells significantly greater selective advantage, everything else being equal. It can be observed in Figure 6 that even thirty-fold increase in the rates of glucose uptake by the glycolytic cells does not make much of a difference in terms of when exactly the rapid increase in the mean of 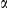 will occur. However, it does raise the maximum value that is reached at higher glucose concentrations. This suggests that upregulation of glucose transporters in glycolytic cells is an adaptation rather than the driving force behind Warburg effect, and therefore therapies targeting glucose transporters would probably not be very effective.

### Modeling evolutionary suicide

**Figure 7:**
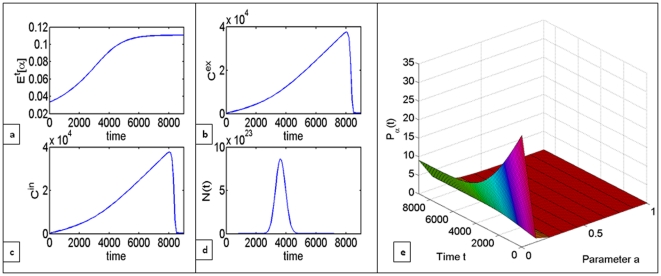
Evolutionary suicide can occur when the proportion of glycolytic cells 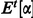 within the total cell population reaches approximately 10% under given parameter values. Trajectories depict (a) the changes in the mean value of glycolytic cells in the population 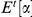 (b) extracellular carbon 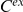, (c) intracellular carbon 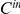, (d) total population size 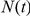 over time and (e) the distribution of cell clones 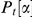 changing over time.

Until now we have been focusing only on the question of whether the increased availability of nutrients can in fact allow the population of glycolytic cells to expand despite the metabolic inefficiency of glycolysis. Now, we would like to consider a case when the increased number of glycolytic cells in the population yields enough lactic acid to be toxic to aerobic cells. This is accounted for through adding an extra death term to the equation that describes the dynamics of the cell population, as well as an additional inflow term in the equation for the changes in the concentration of extracellular carbon, accounting for carbon that is recycled through cell death. On Figure 7 one can see that under given parameter values, the population initially increases in size, but as the proportion of glycolytic cells reaches 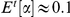, the toxicity from lactic acid becomes higher than cell growth rates. This can be interpreted as the cells committing evolutionary suicide through being overly efficient competitors.

## Discussion

From the point of view of game theory, tumor cells are playing a game of prisoner's dilemma both with somatic cells and with each other. If there are no limitations on oxygen availability, i.e., no severe pressure to choose one metabolic strategy over the other, then the payoffs for aerobic and glycolytic cells are measured in terms of efficiency of metabolism (getting more energy per same amount of glucose) and competitive ability (creating a microenvironment that will be toxic to competitors). It two cells are playing the game of prisoner's dilemma, then one can see using aerobic metabolism as “defecting” and glycolytic as “cooperating” – the cells will get the competitive advantage only if enough of them cooperate. However, the stable equilibrium for the game of prisoner's dilemma is for both players to defect, i.e., for all cells to use aerobic metabolism.

In this particular case one cannot change intrinsic payoffs for the players, i.e., the amount of ATP that each cell receives when it metabolizes glucose aerobically or glycolytically. However, one can change the environment in which they interact in such a way as to minimize the drawbacks of using the “cooperative” strategy. One such way is to supply enough resources for the anaerobic cells to not be held back by the inefficiency of glycolysis.

In order to investigate whether increasing the amount of available nutrients can in fact push the cells out of the stable equilibrium, a mathematical model is proposed to track the change in composition of a parametrically heterogeneous population with respect to the choice of metabolic strategy, i.e., aerobic or glycolytic metabolism. The model is a three dimensional system of ordinary differential equations based on a mathematical model of a chemostat system [20]. There are three state variables that are being kept track of: concentration of extracellular carbon, which is constantly replenished from some external source and is consumed based on difference of concentrations between extra and intracellular concentrations; concentration of intracellular carbon, which is metabolized more efficiently by aerobic cells; and a heterogeneous cell population composed of aerobic and glycolytic cells. The growth of aerobic cells is modeled in such a way as to be constrained both by carbon and oxygen availability. The growth of glycolytic cells is restrained solely by carbon. Parametric heterogeneity within the system is captured by assuming that each cell clone is characterized by an intrinsic value of parameter 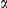, which can range from 0 to 1. The initial distribution of cell clones is assumed to be truncated exponential on the interval 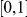, skewed towards 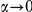 such that a vast majority of clones in the initial cell population are aerobic. The change in population composition is tracked through the change in the mean value of the parameter 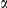, which in this formulation becomes a function of time and thus changes as the system evolves.

Through computation of numerical solutions one could observe that increased inflow of extracellular carbon did indeed cause dramatic changes in the composition of cell population over time (Matlab code is available upon request). However, in order to see any changes in the composition of cell population, glycolytic cells had to have higher growth rates, even if only slightly. This suggests that while increased nutrient availability cannot induce glycolytic switch, it can accelerate disease progression. Decreases in oxygen availability in nutrient-limited environment caused as much of a glycolytic expansion as did dramatic increases in external carbon inflow in normoxic conditions (Figure 4). It was also demonstrated that lower death rates actually slowed down tumor progression at this stage of tumorogenesis because of slower cell turnover rates; increases in death rates caused dramatic increases in the rate of glycolytic expansion because of increased cell turnover (Figure 5), which suggests that cytotoxic therapies would in fact speed up cancer progression. Finally, the effects of differences in resource uptake rates were evaluated, revealing that even 30-fold increases in carbon uptake rates by glycolytic clones do not have nearly as much effect on the rate of glycolytic expansion as do increases in external nutrient inflow.

### The two games

Staying within the aerobic-aerobic equilibrium of the metabolic prisoner's dilemma keeps the tumor (at least temporarily) from switching preferentially to glycolysis, which would lead to creating toxic microenvironment and facilitating metastatic invasion [9], [21]. However, if the environment is changed enough, cells can push away towards glycolytic-glycolytic strategy (everything else being equal), eventually entering the domain of attraction of the stable equilibrium of another, larger game, which can lead to evolutionary suicide [22]. Now glycolytic cells that have become numerous enough are cooperating, jointly increasing the toxicity of the surrounding microenvironment, and becoming more efficient competitors as a group, eventually killing the host and consequently killing themselves.

In the model, this is captured through introduction of the additional toxicity term that captures increased mortality of aerobic cells proportional to the amount of lactic acid secreted by glycolytic cells. Indeed, one can observe that the cell population initially grows, peaks and then eventually collapses, going to extinction (see Figure 7). So, the either equilibrium within the same game of prisoner's dilemma can become attracting not because of the changes in payoffs for each cell but due to different initial composition of the population of players, which happens solely through natural selection.

### Tumors as complex adaptive systems

One way to look at tumors is through the lens of complexity science. Complex systems are diverse and adaptive, and all parts within them are interconnected and interdependent [23]. Tumors fit this definition: they are composed of genetically heterogeneous cells; they are interconnected and interdependent, competing for resources and space with each other and with somatic cells; and they are very adaptable to changes in their microenvironment.

Complex systems are not nearly as predictable as just complicated systems (the ones that have all the characteristics of complex systems except adaptability). They are robust, and they can generate such phenomena as tipping points, which are thresholds of rapid phase transitions [23]. For instance, in the proposed system, changes in the cell microenvironment induced selection for the “cooperative” glycolytic metabolic strategy, which can be viewed as an example of such a tipping point. This can lead to a rapid increase in the amount of lactic acid produced, which in turn can lead to a sudden increase in metastatic spread of the disease due to increased degradation of the extracellular membrane [9]. On a larger scale, one can think of cachexia, nutritionally irreversible loss of body mass, which is often observed in terminal cancer patients, as an example of such a tipping point.

**Figure 8:**
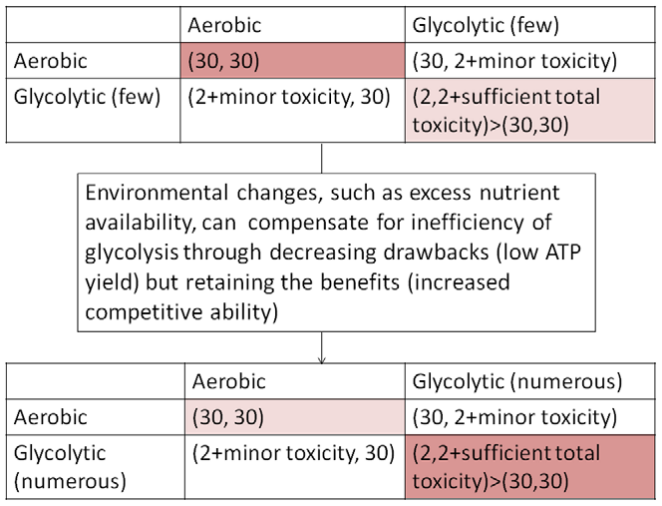
Changes in the microenvironment can lead to changes in population composition, which in turn can lead to the population evolving away from the dominant aerobic-aerobic “defecting” strategy, which keeps the system stable, to glycolytic-glycolytic “cooperation”, which can eventually lead to evolutionary suicide (cancer killing the patient and thus killing itself).

Complex systems cannot be controlled but they can be harnessed, that is, even if one cannot change the intrinsic properties (or in case of game theory, payoffs) of the individual clones, or agents, in the complex systems, one can sometimes change the microenvironment in such a way as to direct system evolution in the desired direction (create an environment, where the players will “want” to choose the strategy that we want them to choose rather than try to force them to do so). For instance, in the metabolism experiment described here, it is the changes in the nutrient availability that enabled the shift within the system towards an otherwise unstable equilibrium (persistence of glycolytic metabolism) by decreasing the negative effect of glycolysis, i.e. low ATP yield, but keeping all of its benefits, i.e., better competitive ability (Figure 8).

Reversing the changes that occurred as a result of surpassing a tipping point in complex systems is usually not possible because of the changes that will have already occurred to the population composition. That is, it is no longer the same “set of players” that is interacting, and therefore their threshold is most probably different. However, tipping points can be anticipated and sometimes even delayed. For instance, several prospective studies have shown that mortality from cancer was much lower in those individuals that had higher muscle mass, regardless of their body mass index (BMI), even though the incidence of cancer was the same (see, for instance, [24], [25]). From the point of view of cell metabolism, this could be due to the fact that muscle cells have higher energy demands than other somatic cells, thus “beating” the glycolytic cells to the nutrients, delaying progression of the disease. So, while exercising will not affect the probability of the person getting cancer in the first place, it may reduce the risk of dying from it by pushing off the metabolic tipping point, surpassing which leads to cancer progression.

### Conclusions

Tumors are complex adaptive systems that consist of a large number of diverse, interconnected and interdependent cells that compete for space and nutrients both with the somatic cells and with each other. One of the measures of tumor diversity could be the type of metabolic strategy that the cell uses for converting glucose to energy: aerobic metabolism has a higher ATP yield and can be seen as an evolutionarily stable metabolic strategy, while glycolysis has a lower ATP yield but it increases the cells' competitive abilities through creating a toxic microenvironment. Tumor cells upregulate glycolysis even in the areas of ample oxygen supply (Warburg effect). It is hypothesized that the benefits of increased acidity of the microenvironment give a large enough payoff to glycolytic cancer cells to overcome the inefficiency of glycolysis. However, glycolytic cells can get this advantage only if enough of them simultaneously use this strategy.

While it is not possible to change the intrinsic energetic payoffs for these cells, changing the microenvironment through providing increased amounts of nutrients can achieve this by decreasing the negative effects of glycolysis (compensating for low ATP yield by providing more carbon) without affecting the benefits (increased competitive ability through elevated lactic acid production). Here we demonstrate that while availability of excess nutrients cannot induce the glycolytic switch, it facilitates disease progression when some glycolytic cancer cells are already present in the population.

It is a common viewpoint that somatic cells always cooperate and cancer cells are the ones that defect, rebelling against cell cooperation within the tissue. However, from the point of view of game theory, choosing aerobic metabolism is in fact a stable “defect-defect” equilibrium in the multi-player game of prisoner's dilemma. And it is the dominance of the defecting strategy that stabilizes the tissue, preventing (as long as possible) occasional glycolytic cooperators from committing evolutionary suicide.

## Supporting Information

Appendix S1 (PDF)

Appendix S2 (PDF)
