# Supplementary material for: Prisoner's Dilemma in Cancer Metabolism
Source: PLoS One. 2011 Dec 14;6(12):e28576. doi: 10.1371/journal.pone.0028576 (PMC3237466; doi:10.1371/journal.pone.0028576)
Supplement: Appendix S1 — (PDF) [file pone.0028576.s001.pdf]

Appendix S1

S1.1 Model Derivation

For all expressions of the type $\frac{\sum_{\mathbb{A}} x_\alpha f(\alpha)}{N}$ we use the standard notation of the expected value $E^t[f(\alpha)]$ of the function $f(\alpha)$ over the distribution $P_\alpha(t) = \frac{x_\alpha(t)}{N(t)}$, where $N(t) = \sum_{\mathbb{A}} x_\alpha(t)$ is the total size of the population if the number of possible values of α is finite and $N(t) = \int_{\mathbb{A}} x_\alpha d\alpha$ if it is infinite.

S1.1.1 Equation for the dynamics of cell clones.

The dynamics of clones x_α is governed by the following equation

$$\frac{dx_\alpha}{dt} = x_\alpha(r_a(1-\alpha)(\frac{\beta C^{in}}{\beta + C^{in}}) + r_g \alpha C^{in} - d - b_\alpha r_g C^{in} E^t[\alpha]) \tag{1}$$

where the growth rate of glycolytic clones α is limited only by availability of intracellular carbon C^{in}, and the growth rate of aerobic clones $(1-\alpha)$ is limited both by carbon C^{in} and oxygen β (for the purposes of this model oxygen inflow is constant and can thus be modeled as a parameter). Cells also die at some constant average rate d.

Incorporating effects of toxicity of lactic acid on the non-glycolytic cell clones is accounted for through the additional death term $b_\alpha = B - b_1\alpha$, where B and b_1 are constants, and where $b_1 < \frac{B}{\alpha}$ due to restriction on $\alpha \in [0,1]$. This functional form incorporates the assumption that the more lactic acid the cell produces, the less sensitive to its toxicity it is (since the clones that produce lactic acid and then immediately die from it cannot persist in the population). Hydrogen transporters are also up-regulated in cancer cells to pump out the acid from the cell.

The total amount of lactic acid released per cell is accounted for by the term $\frac{\sum_A r_g \alpha x_\alpha C^{in}}{N(t)} = r_g C^{in} E^t[\alpha]$, and the cells of type $(1-\alpha)$ that are killed by lactic acid are accounted for with $b_\alpha x_\alpha r_g C^{in} E^t[\alpha]$.

S1.1.2 Equation for the dynamics of extracellular carbon.

The concentration of extracellular carbon that is available for the cells to consume is $C^{ex}_t = C^{ex} N(t)$, where C^{ex}_t is the absolute number of moles of carbon available, C^{ex} is concentration of carbon per cell clone. The inflow of carbon from the blood stream is accounted for with the term $g_1(C_0 - C^{ex})$, where

C_0 is the gradient constant, and g_1 is the rate of inflow-outflow. The term is chosen based on standard models that deal with modeling chemostat.

Extracellular carbon C^{ex} is absorbed by the cells and is converted to intracellular carbon C^{in} at the rate $p(C^{ex} - C^{in})\frac{N(t)}{k_1+C^{ex}-C^{in}}$, which accounts for diffusion of carbon through the cell wall based on the difference between intra- and extracellular carbon concentrations; the limitation $(k_1 + C^{ex} - C^{in})$ is to account for the fact that there is only that much carbon that a cell can absorb. Parameter $p = p_a(1 - \alpha) + p_g(\alpha)$, allowing to take into account the difference in glucose uptake rates between aerobic and glycolytic cells. There is also an inflow of carbon that was released from the cells that died either naturally or due to being killed by lactic acid, coming from the equation for x_α.

So,

$$\frac{d\ C_t^{ex}}{dt} = \frac{dC^{ex}}{dt}N + C^{ex}\frac{N'}{N} = \tag{2}$$

$$= g_1(C_0 - C^{ex}) - p(C^{ex} - C^{in})\frac{N(t)}{k_1 + (C^{ex} - C^{in})} + cC^{in}\sum_{\mathbb{A}} x_\alpha(d + b_\alpha r_g C^{in} E^t[\alpha]). \tag{3}$$

However, when the population of cells is small, which is the focus of the question, for which this system was proposed, the increase in the total population size N' is negligible with respect to the entire population. Therefore, in this case $C^{ex}\frac{N'}{N} \to 0$, yielding the following equation for the change in the concentration of extracellular carbon:

$$\frac{dC^{ex}}{dt} = g_1(\frac{C_0 - C^{ex}}{N}) - p\frac{C^{ex} - C^{in}}{k_1 + (C^{ex} - C^{in})} + C^{in}d + (C^{in})^2 E^t[b]E^t[\alpha]. \tag{4}$$

S1.1.3 Equation for the dynamics of intracellular carbon

The total amount of intracellular carbon available for the cells of type α is $C_{t,\alpha}^{in} = C^{in}x_\alpha$, where C^{in} is the concentration of carbon per cell. The inflow of carbon into each cell clone x_α is accounted for by the term $p\frac{C^{ex}-C^{in}}{k_1+(C^{ex}-C^{in})}$.

Total amount of carbon consumed by the clone x_α is $s(r_g\alpha + (1-\alpha)r_a)C^{in}$, where s is the conversion factor for exactly how much carbon was actually used. How much of it is going to be used for aerobic or anaerobic metabolism depends on the value of the parameter α.

So, similarly to the equation for C^{ex}, the equation for C^{in} becomes

$$\frac{dC^{in}}{dt} = p\frac{C^{ex} - C^{in}}{k_1 + (C^{ex} - C^{in})} - sC^{in}(r_a\xi(1 - E^t[\alpha]) + r_g E^t[\alpha]) \tag{5}$$

where $p = (p_g E^t[\alpha] + p_a(1 - E^t[\alpha]))$.
