# Supplementary material for: Prisoner's Dilemma in Cancer Metabolism
Source: PLoS One. 2011 Dec 14;6(12):e28576. doi: 10.1371/journal.pone.0028576 (PMC3237466; doi:10.1371/journal.pone.0028576)
Supplement: Appendix S2 — (PDF) [file pone.0028576.s002.pdf]

Appendix S2.

S2.1 Distribution of α

Consider the following system:

$$\begin{cases} \frac{dx_\alpha}{dt} = x_\alpha(\underbrace{r_a(1-\alpha)\frac{\beta C^{in}}{\beta + C^{in}}}_{C^{\text{in}} \text{ and } O_2 \text{ lmited aerobic growth}} + \underbrace{r_g \alpha C^{in}}_{C^{\text{in}} \text{ limited glycolytic growth}} - \underbrace{d}_{\text{death rate}} - \underbrace{b_\alpha r_g C^{in} E^t[\alpha]}_{\text{death rate due to lactic acid toxicity}}), \\ \frac{dC^{ex}}{dt} = \underbrace{g_1(\frac{C_0 - C^{ex}}{N})}_{C^{\text{ex}} \text{ inflow from blood}} - \underbrace{(\underbrace{p_g E^t[\alpha]}_{\text{glycolytic cells}} + \underbrace{p_a(1-E^t[\alpha])}_{\text{aerobic cells}})\frac{C^{ex} - C^{in}}{k_1 + (C^{ex} - C^{in})}}_{\text{total } C^{\text{ex}}\text{uptake based on concentration differences}} + \underbrace{\underbrace{C^{in} d}_{\text{natural cell death}}}_{C^{\text{ex}} \text{ recycled from cell death}} \\ \frac{dC^{in}}{dt} = \underbrace{(p_g E^t[\alpha] + p_a(1-E^t[\alpha]))\frac{C^{ex} - C^{in}}{k_1 + (C^{ex} - C^{in})}}_{\text{total } C^{\text{in}} \text{ inflow based on concentration differences}} - sC^{in}(\underbrace{r_a \xi(1-E^t[\alpha])}_{C^{\text{in}}\text{used aerobically}} + \underbrace{r_g E^t[\alpha]}_{C^{\text{in}}\text{used glycolitically}}). \end{cases} \tag{1}$$

where we assume that each clone is characterized by their own intrinsic value of the parameter α but where there is no detectable toxicity from lactic acid (i.e., a case when $b_\alpha = 0$)

Let us introduce keystone variables $g(t)$ and $q(t)$, such that

$$\begin{cases} \frac{dq(t)}{dt} = r_a \beta \frac{C^{in}}{\beta + C^{in}}, \\ \frac{dg(t)}{dt} = r_g C^{in}. \end{cases} \tag{2}$$

Then $x_\alpha(t)' = x_\alpha(t)((1-\alpha)q'(t) + \alpha g'(t) - \delta)$.

Consequently,

$$x_\alpha(t) = x_\alpha(0) e^{(1-\alpha)q(t) + \alpha g(t) - \delta t} \tag{3}$$

Full population size of cells x_α is then given by

$$N(t) = \int_A x_\alpha(t) d\alpha = N(0) \int_A e^{q(t)-\delta t} e^{\alpha(g(t)-q(t))} P_0(\alpha) d\alpha = N(0) e^{q(t)-\delta t} M_0[g(t) - q(t)] \tag{4}$$

where $P_\alpha(0) = \frac{x_\alpha(0)}{N(0)}$ and where $M_0(g(t) - q(t)) = \int_0^\infty e^{\alpha(g(t)-q(t))} P_\alpha(0) d\alpha$ is the moment generating function (mgf) of the initial distribution of clones $P_\alpha(0)$ within the population. The final distribution

would then be given by

$$P_t(\alpha) = \frac{x_\alpha(t)}{N(t)} = \frac{e^{\alpha(g(t)-q(t))}}{M_0[g(t)-q(t)]} \tag{5}$$

System (1) thus becomes

$$\begin{cases} \frac{dC^{ex}}{dt} = g_1(\frac{C_0 - C^{ex}}{N}) - p\frac{C^{ex}-C^{in}}{k_1+(C^{ex}-C^{in})} + C^{in}d, \\ \frac{dC^{in}}{dt} = p\frac{C^{ex}-C^{in}}{k_1+(C^{ex}-C^{in})} - sC^{in}(r_a\xi(1-E^t[\alpha])\frac{\beta}{\beta+C^{in}} - r_gE^t[\alpha]), \\ \frac{dq}{dt} = r_a\beta\frac{C^{in}}{\beta+C^{in}}, \\ \frac{dg}{dt} = r_gC^{in}. \end{cases} \tag{6}$$

The expected value of α at each time t is calculated through Equation 5:

$$E^t[\alpha] = \int \alpha P_t(\alpha)d\alpha = \int P_0(\alpha)\frac{\alpha e^{\alpha(g(t)-q(t))}}{M_0[g(t)-q(t)]} = \frac{M_0'(g(t)-q(t))}{M_0(g(t)-q(t))}. \tag{7}$$

Let us assume that the initial distribution of the clones within the population is truncated exponential, such that $\alpha \in [0,1]$.

For initial truncated exponential distribution with parameter μ, the moment generating function $M_0(t)$ is

$$M_0(t) = \frac{\mu(e^\mu - e^{g(t)-q(t)})}{(e^\mu - 1)(\mu - (g(t)-q(t)))}, \tag{8}$$

and expected value of α is calculated by

$$E^t[\alpha] = \frac{e^\mu - e^{g(t)-q(t)}(g(t)-q(t)-\mu-1)}{(e^{g(t)-q(t)} - e^\mu)(g(t)-q(t)-\mu)}. \tag{9}$$

The parameter $\mu \gg 0$ to ensure that the initial distribution of clones within the population is skewed towards $\alpha \to 0$, that is, such that the majority of the cells have aerobic phenotype, which is what is to be observed in the normal tissue.

S2.2 Distribution of α when $b_\alpha \neq 0$

Now consider a case, when there is also increased cell mortality from the lactic acid that is produced by the glycolytic clones. This is accounted for through the additional death term $b_\alpha = B - b_1\alpha$, where B and b_1 are constants, and where $b_1 < \frac{B}{\alpha}$ due to restriction on $\alpha \in [0, 1]$, which also incorporates the assumption that the more lactic acid the cell produces, the less sensitive to its toxicity it is (the clones that produce lactic acid and then die from it cannot persist in the population). Hydrogen transporters are also up-regulated in cancer cells to pump out the acid from the cell.

In order to study this model, we need to introduce a third keystone equation in addition to System (2):

$$\frac{dv}{dt} = r_g C^{in} E^t[\alpha]. \tag{10}$$

Then the equation for x'_α becomes

$$x'_\alpha = x_\alpha(q'(1-\alpha) + \alpha g' - d - (B - b_1\alpha)v') \tag{11}$$

Integrating this expression yields

$$x_\alpha(t) = x_\alpha(0)e^{q(t)-dt-Bv(t)+\alpha(g(t)-q(t)-b_1 v(t))}. \tag{12}$$

Full population size is then given by

$$N(t) = N(0)e^{q(t)-dt-Bv(t)} M_0(g(t) - q(t) - b_1 v(t)) \tag{13}$$

and the distribution of clones is given by

$$P_t(\alpha) = \frac{x_\alpha(t)}{N(t)} = \frac{e^{\alpha(g(t)-q(t)-b_1 v(t))}}{M_0(g(t) - q(t) - b_1 v(t))}. \tag{14}$$

The full system thus becomes

$$
\begin{cases}
\frac{dC^{ex}}{dt} = g_1(\frac{C_0 - C^{ex}}{N}) - p\frac{C^{ex}-C^{in}}{k_1+(C^{ex}-C^{in})} + C^{in}d + (C^{in})^2 E^t[b]E^t[\alpha], \\
\frac{dC^{in}}{dt} = p\frac{C^{ex}-C^{in}}{k_1+(C^{ex}-C^{in})} - sC^{in}(r_a\xi(1-E^t[\alpha])\frac{\beta}{\beta+C^{in}} - r_g E^t[\alpha]), \\
\frac{dq}{dt} = r_a\beta\frac{C^{in}}{\beta+C^{in}}, \\
\frac{dg}{dt} = r_g C^{in}, \\
\frac{dv}{dt} = r_g C^{in} E^t[\alpha].
\end{cases} \tag{15}
$$

The proposed system is used to address two different questions: 1) whether the changes in nutrient availability are sufficient to switch population composition towards glycolytic phenotype, and 2) whether excessive toxicity from lactic acid can cause the population to commit evolutionary suicide. For the purposes set out by the first question, parameter b_α is taken to be zero. This is done to be able to more clearly see, whether the shift can in fact occur to a significant enough extent. When $b_\alpha \neq 0$, population extinction due to acid toxicity occurs too quickly (around $E^t[\alpha] \approx 0.1$ for the considered parameter values), and the effects of excess nutrients cannot be evaluated very well.
